# Analysis of Biological Features Associated with Meiotic Recombination Hot and Cold Spots in *Saccharomyces cerevisiae*

**DOI:** 10.1371/journal.pone.0029711

**Published:** 2011-12-29

**Authors:** Loren Hansen, Nak-Kyeong Kim, Leonardo Mariño-Ramírez, David Landsman

**Affiliations:** 1 Computational Biology Branch, National Center for Biotechnology Information, National Library of Medicine, National Institutes of Health, Bethesda, Maryland, United States of America; 2 Boston University, Bioinformatics Program, Boston, Massachusetts, United States of America; 3 Department of Mathematics and Statistics, Old Dominion University, Norfolk, Virginia, United States of America; 4 PanAmerican Bioinformatics Institute, Santa Marta, Magdalena, Colombia; Georgia Institute of Technology, United States of America

## Abstract

Meiotic recombination is not distributed uniformly throughout the genome. There are regions of high and low recombination rates called hot and cold spots, respectively. The recombination rate parallels the frequency of DNA double-strand breaks (DSBs) that initiate meiotic recombination. The aim is to identify biological features associated with DSB frequency. We constructed vectors representing various chromatin and sequence-based features for 1179 DSB hot spots and 1028 DSB cold spots. Using a feature selection approach, we have identified five features that distinguish hot from cold spots in *Saccharomyces cerevisiae* with high accuracy, namely the histone marks H3K4me3, H3K14ac, H3K36me3, and H3K79me3; and GC content. Previous studies have associated H3K4me3, H3K36me3, and GC content with areas of mitotic recombination. H3K14ac and H3K79me3 are novel predictions and thus represent good candidates for further experimental study. We also show nucleosome occupancy maps produced using next generation sequencing exhibit a bias at DSB hot spots and this bias is strong enough to obscure biologically relevant information. A computational approach using feature selection can productively be used to identify promising biological associations. H3K14ac and H3K79me3 are novel predictions of chromatin marks associated with meiotic DSBs. Next generation sequencing can exhibit a bias that is strong enough to lead to incorrect conclusions. Care must be taken when interpreting high throughput sequencing data where systematic biases have been documented.

## Introduction

Meiosis is the biological process by which the genome is divided in half to generate daughter cells that can participate in sexual reproduction. In eukaryotes, this process is accompanied by meiotic recombination, which involves pairing of homologous chromosomes and exchanging of genetic material. Meiosis serves to increase genetic diversity in progeny (for review see [1] and [2]). Recombination does not occur with a uniform frequency across the genome. Instead, there are regions with high and low recombination rates called hot and cold spots, respectively. Recombination is initiated by double-strand breaks (DSBs) which are catalyzed by Spo11 [3]. In this biological event, broken DNA ends are processed to produce single-strand ends that can invade the homologous chromosome [4].

Mapping DSB hot spots [5], [6], [7] and factors correlated with hot/cold spot formation is an active area of research. Several biological features have been found to correlate with higher levels of Spo11-catalyzed DSBs. Genome-wide mapping and analysis of Spo11-catalyzed DSB sites in the yeast *Saccharomyces cerevisiae* showed that regions with a high break frequency had a high G+C content [7]. A recent study using this same dataset revealed that several types of microsatellites were associated with recombination hot spots [8]. Additionally, studies using machine learning-based techniques and sequence-based features have differentiated DSB hot and cold spots somewhat successfully [9], [10], suggesting that differences in sequence composition between these regions exist.

In addition to sequence-based factors, chromatin structure is associated with regions of high and low recombination. Many hot spots exhibit an open chromatin structure constitutively in both meiotic and mitotic cells [11], [12]. Some of these hot spots also show an increase in micrococcal nuclease (MNase) sensitivity in meiotic cells shortly before DSB formation [13], indicating active chromatin remodeling to a more open configuration upon the onset of meiosis. Some posttranslational histone marks are also associated with increased DSB frequency, with H3K4me4 and bulk histone acetylation (in *Schizosaccharomyces pombe*) showing a positive correlation [14], [15] and H3K36 methylation exhibiting a negative correlation. Here we used a multivariate feature selection approach to determine the sequence and chromatin features that best distinguish hot and cold spots in *S. cerevisiae*. The histone modifications and nucleosome occupancy data used in our analysis were derived from vegetatively growing mitotic cells, which is a different cell state than meiotic cells. Genome-wide epigenetic studies using both mitotic and meiotic states were used to increase the amount of useable data; there is good reason to believe that epigenetic marks found at hot or cold spots in mitotic cells will also be present at those same sites in meiotic cells (see Discussion).

Feature selection is a dimensionality reduction technique designed to identify the subset of features that is most informative in producing robust predictive models. Feature selection has been used successfully in microarray gene expression studies [16], [17] and biomarker identification [18], [19]. When attempting to build a classifier based on vectors of features, many features are irrelevant. For example, a common task in microarray studies is to identify which genes are relevant in distinguishing between two or more experimental conditions. In this case, the expression level of thousands of genes (i.e., features) is measured, but only a small subset is relevant in discriminating between the experimental conditions. Many pattern recognition techniques were not designed to deal with circumstances in which the number of relevant features is outnumbered by irrelevant ones [20]. In these instances, feature selection can be used to reduce over fitting, improve predictive performance by identifying a subset of relevant features, and provide insight into the underlying biological processes that generated the data. Machine learning-based approaches have already been applied to the problem of discriminating between hot and cold spots [9], [10]. However, these studies analyzed low resolution data and feature selection was not performed. Here we report the results of applying feature selection to identify factors associated with recombination hot and cold spots. A feature vector as used in this study is a string of numerical features; each feature in the string represents a measurement of a biological quantity.

## Methods

### Definition of hot and cold regions

Buhler *et al.* [5] mapped the frequency of meiotic DSBs in S. *cerevisiae* with high resolution tiling arrays. Using this data, we obtained 1179 and 1028 regions identified as hot and cold spots, respectively, for a total of 2207 regions. Each region was 600 base pairs (bp) in length. Buhler *et al.* produced a set of peaks representing hot spots with 5-fold and 2-fold enrichment over background. In our analysis, hot spots were defined by centering a 600-bp window at the midpoint of peaks that were enriched 5-fold over background. Cold spots were obtained by finding at least three adjacent probes with a log2 hybridization ratio of less than 0.75, and then centering a 600-bp window at the midpoint of the centermost probe. For each region, we produced a vector of length 350 to represent features such as the chromatin-associated factors “Nucleosome occupancy”, “H3K14ac”, “H3K36me3”, “H3K4me1”, “H3K4me2”, “H3K4me3”, “H3K79me3”, and “H3K9ac”.

Pan *et al.* [21] identified hot spots by mapping the binding of Spo11 using high throughput sequencing. We centered 600-bp windows at the middle of hot spots as defined by Pan *et al.* Cold spots were defined by a set of non-overlapping 600 bp windows with no reads aligned that did not overlap to any extent simple repeats as downloaded from the UCSC genome browser.

### Generation of chromatin structure-based features

Pokholok *et al.* used tiling arrays to map histone modifications in S. *cerevisiae*. We obtained this data from the public database ArrayExpress Archive (http://www.ebi.ac.uk/arrayexpress/) and normalized using MA2C normalization [22]. There are a number of publically available datasets containing additional chromatin marks mapped genome wide that potentially could have been included in this study. Unfortunately they are low resolution; one microarray element per ORF or intergenic region or they do not control for differences in nucleosome occupancy. For each region, we obtained the degree of enrichment by averaging the normalized hybridization values of the probes within that region. For example, the feature “H3K14ac” represents the average degree of acetylation of lysine 14 in histone H3 for the given region. A similar approach was used for each histone modification. To calculate the degree of nucleosome occupancy we used a dataset produced by Kaplan *et al.* [23]. For most positions in the genome, Kaplan and co-authors calculated a nucleosome occupancy score. The average nucleosome occupancy was normalized to zero. A value greater than zero represents nucleosome enrichment relative to the genome-wide average, while a value less than zero signifies nucleosome depletion. For each hot or cold region, nucleosome occupancy was calculated by averaging the nucleosome occupancy scores for that region.

### Generation of sequence-based features

In this study, 342 out of 350 features were sequence-based in which each sequence feature represented the normalized frequency of the region for one of the 1–4 possible k-mers. For example, feature 9 for region × would be the number of times the 2-mer “AT” was found in the region divided by the number of k-mers of size 2 found in the region. Hence the feature represents the enrichment of AT relative to all 2-mers found in the region. Similarly, feature 300 for region × would be the number of times “AAGT” was found in the region divided by the number of k-mers of size 4 found in the region. We also included two sequence features “AT content” and “GC content”, reflecting the overall AT and GC content for that region, respectively. It would seem the sequence features could further be reduced by removing the reverse complement of the given k-mer (CG is the same as GC). Whether or not the reverse complement is redundant is based on whether or not strand specific processes are acting at Hot spots. There are examples of strand specific trans-acting factors operating at hot spots [24]. Hence reverse complements were retained in the final set of features.

### Feature selection

Feature selection can be described as finding the subset of features from the set of all possible combinations of features that can best distinguish classes of interest. Because the search space of all possible combinations of features grows exponentially with the number of features, it is rarely feasible to perform an exhaustive search. Instead, various heuristic search methods can be used to identify meaningful feature subsets that can be used to build classifiers with high accuracy. Here we used a genetic algorithm (GA)- based approach [25] similar to those published previously [26], [27], [28]. We used the R package Galgo [29] to implement the algorithm.

**Figure 1:**
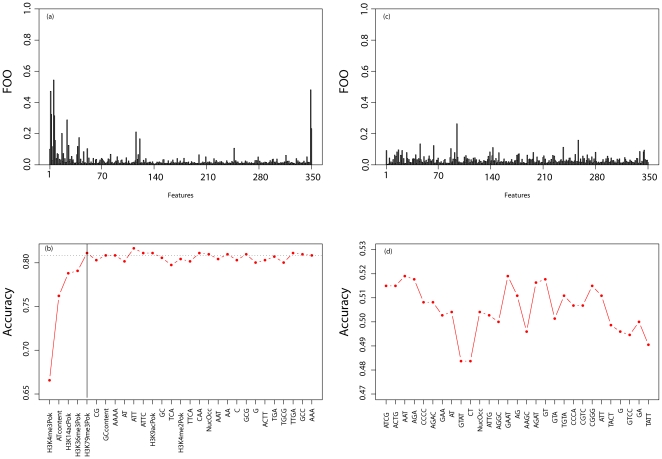
Overview of the feature selection procedure. The initial set of 2207 regions was divided into a training set of 1471 regions and a testing dataset containing 736 regions. The training dataset was further divided into sub-training and validation datasets. (a) The (Genetic Algorithm) GA was run 10,000 times on different sub-training and validation datasets, producing a subset of optimal features for each run (see Methods). We divided the number of times each feature occurred in an optimal feature subset by the total number of times the GA was run (i.e., 10,000) to calculate the frequency of observation (FOO). Features that occurred most often in many different optimal subsets across different splits of the training dataset were ranked higher than features that were selected less often. (b) To obtain the final subset, features were added individually based on their FOO score from highest to lowest. Then, the corresponding accuracy using the testing dataset was calculated. Features were added until no substantial improvement in accuracy was observed, indicated in the figure panel (b) by the solid black line. Panels (c) and (d) are identical to (a) and (b) except random regions were used (i.e., 1179 and 1028 regions randomly selected and labeled as “hot” and “cold”, respectively).

The dataset of 2207 features was divided randomly into two groups, a training dataset containing 1471 regions and a testing dataset containing 736 regions. Each dataset contained roughly equal numbers of hot and cold regions. The training dataset was further divided into three pairs of sub-training and validation datasets. Each pair of the sub-training datasets contained 981 regions, while those of the validation dataset contained 490 regions. The GA was then applied to these datasets in search of a subset of features with optimal accuracy based on the average accuracy across all sets of sub-training and validation data. More specifically, the GA searched for a feature subset that optimized a score defined as A_total_ = (A_1_+A_2_+A_3_)/3, in which A_i_ is defined as the accuracy of the given subset of features using a random forest classifier built utilizing the sub-training dataset *i* and tested on the validation dataset *i* and *i* = {1, 2, 3}. In general, accuracy was defined as the total number of regions classified correctly divided by the total number of regions in the validation dataset. The search space of 350 features was prohibitively large, and running the GA twice on the same training and validation datasets would most likely have yielded two different solutions representing local optima. Thus, a sampling of the fitness landscape was used in which the GA was run 10,000 times on different random divisions of the training dataset into the sub-training and sub-validation datasets. The final solution was obtained by combining the results of these independent runs. Features were ranked according to their frequency of occurrence within the subset of optimal features selected by the GA. Features that were present across many runs were presumed to be more important than those that were selected less often (Figure 1). For example, if feature one was present in 9,000 of the 10,000 optimal subsets returned by the GA, while feature two was present in only 5,000, then feature one would be considered more important and thus ranked higher than feature two. The final subset of features was obtained using a forward selection approach. Features were added individually based on ranking until no significant improvement in accuracy was observed. The corresponding accuracy was calculated using the testing dataset.

### Alignment methodology

Alignments were performed using BLASTN with default parameters [30]. When allowing multimapping of reads we followed the procedure as defined in [31]; briefly any alignment yielding an identify less than 90% was discarded and, for alignments between 90% and 95%, only the maximum score was retained. All alignments with greater than 95% identity were kept. Identity was defined as alignment length divided by read length.

### MNase control subtraction methodology

Normalizing for differences in sequencing coverage was accomplished by dividing read counts at each base pair by the total number of unique mappable reads for each dataset, similar to the procedure used in [32]. The following formula was used to subtract out the normalized counts of the MNase control. Given two sequencing datasets D_1_ and a control D_2_ with normalized counts of read coverage at each base pair represented by c_1_ = {c_1,1_, c_1,2_ ,….c_1,m_} and c_2_ = {c_2,1_, c_2,2_ ,….c_2,m_}, the subtracted read density was defined at each base pair as
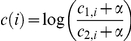
where 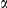 is a constant set to 2 to avoid division by zero errors and to dampen noise.

## Results

A dataset consisting of 2207 regions (1179 hot spots, 1028 cold spots) was first randomly divided such that two-thirds were analyzed by feature selection (see Materials and Methods) and one-third was set aside as a testing dataset. The testing dataset was used to test how accurately the features identified can distinguish between hot and cold spots. Setting aside a testing dataset ensures a fair test with the features being tested on data not used to obtain the features. Hot spots as used in this manuscript refer to regions of increased meiotic DSBs and cold spots to regions of decreased meiotic DSBs. Features were first ranked in order of importance based on the training dataset. The final subset of features was obtained using a forward selection approach. Features were added individually based on ranking until no significant improvement in accuracy was observed. The corresponding accuracy was calculated using the testing dataset. Thus, accuracy using only highly ranked features was estimated based on data not used to rank the features. A subset of five features (i.e., H3K4me3, H3K14ac, H3K36me3, H3K79me3, and GC content) was identified (Figure 1) with a classification accuracy of 80.4%, sensitivity of 80.5%, and specificity of 80.3%. Many of the identified features were found to be associated with recombination, according to published literature.

### Chromatin Structure

**Figure 2:**
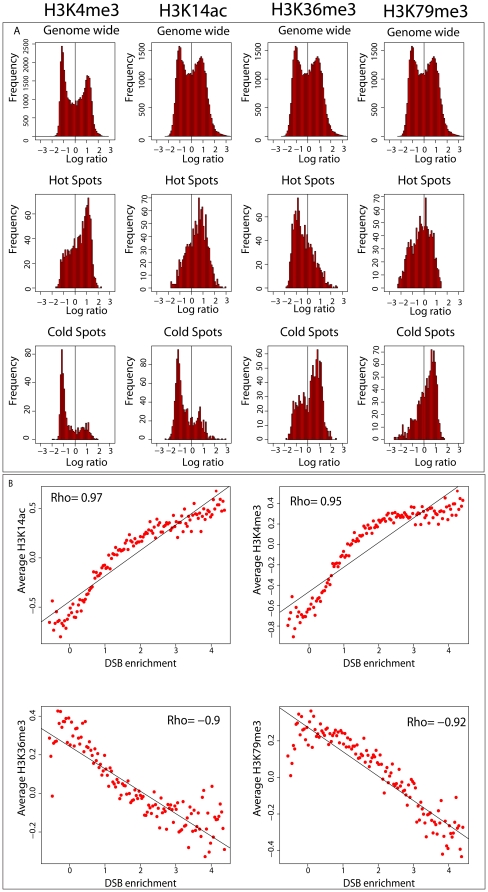
Selected histone marks are correlated with meiotic DSB frequency. (A) Presence of histone marks at hot or cold spots. The first row displays histograms of the log ratios for all probes on the microarray. The higher the log ratio, the more enriched is the given mark. The second row is the enrichment of the histone marks at hot spots. Log ratios were binned in 600-bp windows centered at hot spots and the averages for each bin plotted. The third row is the enrichment of the histone marks at cold spots. Log ratios were binned in 600-bp windows centered at cold spots and the averages for each bin plotted. (B) Histone mark enrichment is correlated with DSB frequency. Probes on both microarrays measuring DSB enrichment and histone modification were paired based on whether they mapped to the same genomic location. Pairs of probes were then grouped in 100 bins according to their DSB enrichment (x-axis). The corresponding log ratios measuring histone modification for the given mark were then averaged for the probes in each bin (y-axis). Bins representing extreme DSB enrichment values had a very low number of probes ∼1–10 hence the histone modification averages for these bins was highly variable. Therefore any bin containing less than 50 probes was discarded.

All of the histone modifications used in this study were mapped in vegetatively growing mitotic cells. While the DSB frequency dataset used to map meiotic hot and cold spots was obtained from meiotic cells we address this issue in more detail in the discussion section. The feature selected as having the highest predictive importance was the degree of H3K4me3 methylation. Published literature strongly associates this mark with recombination hot spots. In *S. cerevisiae*, the methyltransferase Set1 is responsible for H3K4 methylation. Set1 mutants exhibit dramatically reduced DSB frequency at well-characterized hot spots [33]. Additionally, H2B ubiquitination promotes Set1 activity [34], thereby increasing H3K4 methylation. Preventing this mark leads to decreased DSB frequency [35]. Importantly, Borde *et al.* [14] demonstrated that deleting Set1 reduced or eliminated DSBs at 84% of the hottest sites in S. *cerevisiae.* In addition, recent work has associated PRDM9, a sequence-specific DNA binding methyltransferase, with hot spot activity in mammalian meiosis [36], [37], [38]. Our results are consistent with these studies, indicating that H3K4me3 associates positively with areas of high recombination (Figure 2).

**Figure 3:**
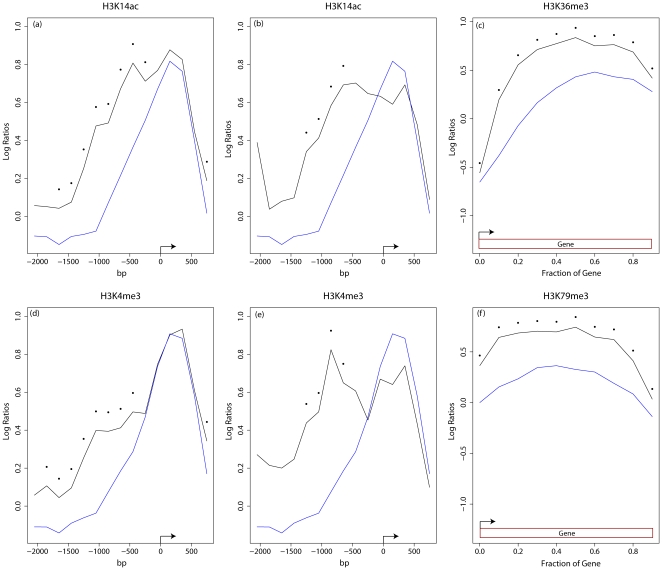
Plots of average modification level around transcription start sites (TSS). The x-axis represents- position relative to the TSS set at zero. Positive numbers represent positions downstream of the TSS, while negative numbers are upstream. The y-axis indicates the average histone modification enrichment log ratios. Black dots represent points statistically significantly different (p-value<0.01 wilcox rank sum test) than the corresponding point in the other curve. Forpanels (a, b, d and e) the blue line represents TSS at least 3000 bps away from the center of a hot spot, log ratios were binned in 200-bp windows and the average for each bin plotted. The black line represents genes with the center of a hot spot located within 500 bp upstream of the TSS,log ratios were binned in 200-bp windows and the average for each bin plotted. For panels (c and f) the black line represents the average histone modification in genes which entirely contain a cold spot (for definition of cold spot see Methods). The blue line represents the average histone modification in genes which do not overlap to any extent a cold spot. Plots were produced by binning histone modification log ratios in bins proportional to gene size (each bin was 1/10 the size of the given gene) the average for each bin is plotted.

H3K14ac is a histone mark associated with active transcription. Like H3K4me3, H3K14ac is localized primarily to the 5′ end and promoter region of open reading frames and is correlated with the rate of transcription [39], [40], [41]. Research has linked histone acetylation with meiotic DSB frequencies. For instance, Sir2 deacetylates histones H3 and H4 [42]. Mutants deficient in Sir2 exhibit widespread changes in meiotic DSB frequencies with 12% of yeast genes showing altered DSB frequency [43]. Moreover, the histone deacetylase Rpd3 represses meiotic recombination at the well-studied hot spot *HIS*4 in S. *cerevisiae* [44]. Finally, deletion of the histone acetyltransferase *GCN*5, which preferentially acetylates H3 histones, leads to decreased recombination at the *ade6-M26* hot spot in S. *pombe* [15]. Our analysis indicates that H3K14ac is associated with DSB hot regions, with high levels of this mark corresponding to hot spots and low levels to cold spots (Figures 2 and 3).

H3K36me3 is a post-translational modification catalyzed by the methyltransferase Set2, and is found primarily in the coding region of genes being actively transcribed [39], [40]. By recruiting the repressor Rpd3, H3K36me3 suppresses spurious transcription initiation [45]. H3K36me3 may also play a role in differentiating exons from introns [46]. Our results indicate that the presence of H3K36me3 may play a largely inhibitory role in DSB frequency as this mark is enriched in cold spots relative to hot spots (Figures 2 and 3). In addition, studies have shown that Set2 the methyltransferase responsible for H3K36me3 represses meiotic recombination at the HIS4 hot spot in yeast [44].

Like H3K36me3, H3K79me3 is found primarily within coding regions. Unlike H3K36me3, however, the degree of H3K79me3 presence is not strongly associated with transcription [40]. The exact function of this mark is unknown, although some evidence suggests that H3K79me3 may play a role in histone H3 exchange [47]. Our results indicate H3K79me3 may play a minor repressive role in DSB frequency since cold spots appear to be enriched for H3K79me3 (Figures 2 and 3). Most of the histone modification features show a strong partitioning with hot spots being either enriched or depleted for the chromatin mark and vice versa for cold spots. H3K79me3 is an exception cold spots are enriched for this mark but hot spots are not depleted instead showing about the genome average of H3K79me3 (Figure 2 panel a). This trend could be explained by H3K79me3 having a lesser effect on DSB frequency or by an indirect effect.

Computational analysis is rarely capable of demonstrating a causal relationship. Feature selection can identify which biological features out of a large number of candidate features are associated with regions of high/low meiotic DSBs. The method cannot identify the reason behind the association. Once an association is discovered it is important to identify potential confounding variables and test whether they may be solely responsible for the correlation of biological features. Such an analysis cannot prove a causal relationship but it is helpful in elucidating uninteresting correlations.

An important confounding variable that arises when working with recombination hot spots is their tendency to localize to promoter regions while cold spots localize to coding regions. Many of the histone marks we studied also have a tendency to localize either to the 5′ end of genes or to coding regions. Therefore, it is possible that the results of our analysis reflect this co-localization effect. To explore this, we compared promoter regions of genes with a hot spot within 500 bp upstream of the transcription start site (TSS) (N = 218) to those genes whose TSS is at least 3000 bp away from a hot spot (N = 2491) (Figure 3 panels a and d). Divergent promoters were removed from this analysis. Gene coordinates were obtained from the UCSC genome browser.

Both H3K14ac and H3K4me3 exhibit a “peak” of modification in promoters of genes that contain hot spots. This “peak” is absent in promoters that lack hot spots. H3K14ac and H3K4me3 are positively correlated with transcription. It is possible that the enrichment of H3K14ac and H3K4me3 observed upstream of genes close to hot spots is due to increased transcriptional rates. To test this we obtained gene expression data [48] and compared transcription rates. The set of genes with a hot spot upstream of the TSS, on average do have a higher transcriptional rate compared with genes whose TSS is at least 3000 bp away from a hot spot (2.2 mRNA/h compared to 1.7 mRNA/h, p-value = 0.003, Wilcox rank sum test), an association that has previously been reported [7].

To test whether this difference in transcription could explain the extra enrichment of H3K14ac and H3K4me3 upstream of the TSS we plotted these marks for genes with an upstream hot spot whose transcriptional rate was less than 1 mRNA/h (Figure 3 panels b and e) (N = 43). The peaks of upstream enrichment are retained even for inactive genes. This analysis indicates that H3K14ac and H3K4me3 enrichment in areas of high recombination is likely not due solely to the tendency of hot spots to localize to promoter regions or to differences in transcriptional activity. Similarly, we compared coding regions that entirely contain a cold spot to those that do not overlap to any extent with cold spots (Figure 3, panels c and f). Genes that contain cold spots show an increased enrichment for both H3K36me3 and H3K79me3. Both H3K36me3 and H3K79me3 within gene bodies are positively correlated with transcriptional activity [40], H3K36me3 is strongly correlated and H3K79me3 is weakly correlated. Perhaps the increased enrichment of H3K36me3 and H3K79me3 in genes containing cold spots compared to genes without cold spots is due to the fact that cold spots are preferentially located in active genes. We compared transcriptional rates for genes with (N = 498) and without cold spots (N = 4516). Genes with cold spots have lower transcription rates than genes without cold spots (median transcriptional rate 1.3 mRNA/h compared to 2.3 mRNA/h, p-value<1e-16 Wilcox rank sum test). Even though genes containing cold spots have on average lower transcriptional rates than genes without cold spots they exhibit a higher degree of H3K36me3 and H3K79me3 methylation (Figure 3 panels c and f).

Holstege *et al.* measured gene expression in mitotic cells. The purpose behind the preceding analysis is to check whether the observed patterns of histone modifications at hot or cold spots are due to differences in gene activity and not to the presence or absence of a hot or cold spot. Given that the histone modifications were measured in mitotic cells, the appropriate dataset for the above analysis is gene expression also measured in mitotic cells. While this manuscript was in preparation, a high resolution map of DSB hot spots was published [21]. This map was produced by sequencing and mapping oligos bound by Spo11 where the hot spots were mapped at much higher resolution than the Buhler *et al.* dataset. We obtained the set of hot spots mapped by Pan *et al.* in order to check if the association of meiotic DSB frequency with the histone marks H3K14ac, H3K4me3, H3K36me3 and H3K79me3 observed using the Buhler *et al.* dataset were also observed using an independently produced higher resolution hot spot map. The Pan *et al.* hot spots, like the Buhler *et al.* hot spots, strongly localized to promoter regions [21]. Hence, a positive correlation with H3K14ac and H3K4me3 and a negative correlation with H3K36me3 and H3K79me3 would be expected.

We duplicated the analysis described in Figure 3 using the Pan *et al.* hot spots, and found similar results to what was seen using the Buhler *et al.* hot spots. Additionally, we show that the H3K14ac and H3K4me3 peaks observed upstream of genes with a hot spot are in general proportional to the strength of the hot spot (Figure S1). The comparison of gene expression rates between hot spot associated genes and non-hot spot associated genes and cold spot associated genes with non-cold spot associated genes was performed using gene expression obtained in vegetatively growing mitotic cells. To check if the same patterns are observed with meiotic cells we repeated the above comparisons with gene expression measured at different time points after cells were placed in sporulation media (Figure S2) gene expression data was taken from [49]. The expression dataset used measured gene expression for four yeast strains SK1, non-sporulating SK1 control, W303 and a non-sporulating W303 control. For the non-sporulating controls which do not enter meiosis the above described patterns held true for all time points. That is hot spot associated genes are transcriptionally more active than non-hot spot associated genes and cold spot associated genes are transcriptionally less active than non-cold spot associated genes.

Interestingly, this pattern did not hold true in the case of hot spot genes compared to non-hot spot genes in meiotic cells. Upon the entrance to meiosis the difference in gene expression between hot and non-hot genes gradually falls to zero (Figure S2 panel's b and d). This could be explained by the observation that hot spot associated genes have a tendency to be repressed in meiosis [7]. Cold spot associated genes are transcriptionally less active than non-cold spot genes in both mitotic and meiotic cells (Figure S2 panel's e, f, g and h).

As discussed above there is ample evidence from multiple studies that H3K4me3 is involved in hot spot selection. Given that histone marks are in general correlated with one another [39], is it possible the association of H3K14ac, H3K79me3, and H3K36me3 with DSB frequency is simply a consequence of these marks being correlated with H3K4me3? In the case of H3K36me3 there is previous research linking this mark with hot spot activity at a well-studied hot spot in yeast [44]. As discussed above multiple studies have linked histone acetylation with hot spot activity.

H3K4me3 in general is correlated with other histone marks but it is particularly strongly correlated with H3K14ac (r = 0.85, p-value<2.2 e-16) compared to its correlation with H3K4me2 which is the next strongest correlation (r = 0.62, p-value<2.2 e-16). Even when comparing a large number of histone marks H3K4me3 is inordinately strongly correlated with H3K14ac [39]. Taken together with the previous work linking histone actylation with recombination, the usually strong correlation of H3K4me3 with H3K14ac combined with our results suggests these marks may act together at meiotic DSB hot spots. While there is a statistically significant correlation between H3K4me3 and H3K79me3 (r = 0.09, p-value<2.2 e-16) this correlation is too small and in the wrong direction to explain the association of H3K79me3 with meiotic DSB frequency.

### AT/CG Content

One of the features selected by the feature selection algorithm was a sequence based feature AT content. AT content and GC content measure the same quantity and both were included in the input feature set as a “sanity check” or control. If our computational method is working correctly, then these features should rank similarly. Indeed, this is what was observed AT content ranks 2^nd^ out of 350 features GC content ranks 7^th^ (Figure 1) . Our analysis is in agreement with published results [7] indicating that GC content in hot spots is higher than the overall average in S. *cerevisiae*. More specifically, the mean GC content within a 600-bp window centered on hot spots was 39.6%, while the GC content of the entire genome was 38.1%. Not surprisingly, the mean AT content in cold spots (63.8%) is greater than that across the entire genome (61.9%).

To further explore the relationship between GC content and recombination cold spots we examined the set of cold spots found entirely within coding sequences. Coding sequences in yeast have a GC content of 39.6%, which is GC rich relative to the genome as a whole. The mean GC content of cold spots found entirely within coding sequences was 37.0% compared to the genome average of 38.1% and compared to 36.0% percent GC content calculated for the entire set of cold regions. Cold spots found within otherwise GC-rich regions (i.e., coding sequences) still showed reduced GC content contrary to the overall trend of coding regions as a whole. Studies have shown that hot spots are generally absent from protein coding sequences despite their high GC content [50], [51]. Our results suggest that cold spots may be associated with regions of low GC and high AT content within coding sequences.

### Nucleosome Occupancy

Of the four biological features included in our analysis with previous evidence from the literature associating them with meiotic DSBs (H3K4me3, H4K36me3, GC content and nucleosome occupancy) three were selected by our method (H3K4me3, H3K36me3, GC content). Our method did not identify nucleosome occupancy as an important feature distinguishing hot from cold spots. This is surprising since multiple studies [11], [12], [52] have suggested that recombination hot spots are typically found in regions of increased sensitivity to nucleases, presumably reflecting a local open chromatin structure. The dataset we used to test nucleosome occupancy was produced by Kaplan *et al.* [23] and based on high throughput sequencing technology.

**Figure 4:**
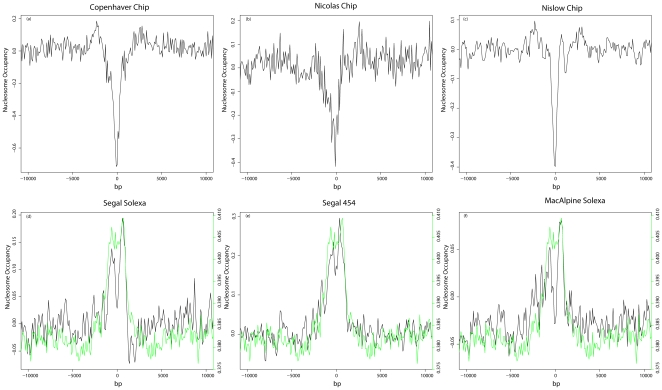
Nucleosome occupancy at hot spots. Multiple nucleosome occupancy maps produced using three different technologies (i.e., FAIRE, Chip-Chip, Chip-Seq) were obtained. Hot spots were aligned Z-score standardized nucleosome occupancy as is shown in 100 bp bins (y-axis). The center of the aligned hot spots is zero on the x-axis. (a–c) Nuclesome occupancy maps based on microarray technology. The sign was reversed in panel a to be consistent with how nuclesome depeletion is represented in the other microarry-based techniques. (d–f) Nuclesome occupancy maps based on high throughput sequencing. The green line plots the mean GC content around hot spots as calcuated by averaging the GC content in 100-bp bins. The y-axis scale on the right is for the GC content plot. The first word in each plot title is the last author on the paper in which the given dataset was described. (references for datasets: a [54] , b [14], c [55], d [23], e [76], and f [77]). Nucleosome occupancy scores were used as calculated by the authors.

One possible explanation for our results is that chromatin remodeling may be occurring after cells have entered meiosis. Kaplan *et al.* measured nucleosome occupancy using data derived from vegetatively growing mitotic cells. There are examples of hot spots showing a closed chromatin structure during mitosis but an open one in meiosis [53]. However, a recent study that measured nucleosome occupancy using formaldehyde-assisted isolation of regulatory elements (FAIRE) showed that meiotic DSB hot spots genome-wide overlapped with nucleosome-free regions in mitotic cells greater than would be expected by random chance [54] which greatly weakens the above hypothesis. To investigate this further, we obtained a set of nine different nucleosome occupancy maps from three microarray-based and six high throughput sequencing-based studies and examined nucleosome occupancy around hot spots in each dataset. All six sequencing-based datasets are plotted together in Figure S3. All of the sequencing based datasets fragmented DNA using nuclease digestion. Two of the microarray based nucleosome positioning maps used sonication. One of them, (Figure 4 (c)) similar to the sequencing based datasets used micrococcal nuclease digestion [55]. The Lee *et al.* dataset also mapped nucleosome positions at a high resolution ∼4 bp similar to the 1 bp resolution of the sequencing based studies. Our analysis yielded a discrepancy in the results comparing microarray- and sequencing-based nucleosome occupancy maps. The microarray-based results all show a well-defined valley representing nucleosome depletion centered at hot spots. Based on these results and previously referenced studies, we conclude that the microarray results best approximate what occurs *in vivo*. On average, nucleosomes are depleted at hot spots for mitotically dividing cells. Contrary to these results, the sequencing-based datasets yielded a small peak of nucleosome occupancy at hot spots (Figure 4). Some datasets exhibited a variable amount of bias (compare peak to baseline differences Figure 4 panels d and e to Figure 4 panel f).

**Figure 5:**
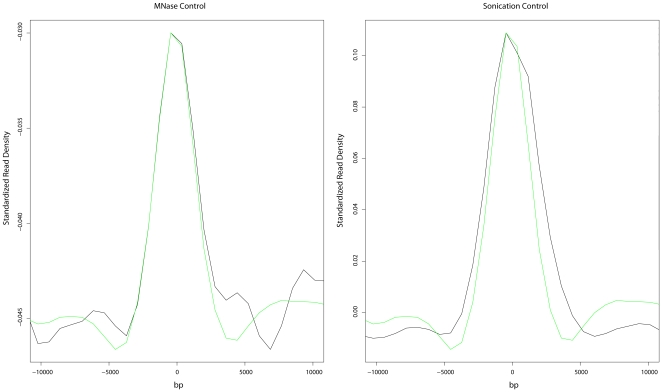
Read density for sequencing controls at hot spots. (a) Purified DNA digested with micrococcal nuclease (MNase) and sequenced using the Solexa platform. (b) Purified DNA following sonication and sequencing using the Solexa platform. The black line indicates the z-score standardized mapped read density, while the green line depicts GC content as calculated in Figure 4. Data was smoothed using loess smoothing.

We obtained and plotted read density at and around hot spots using two publicly available control datasets (Figure 5). Control dataset “a” was produced by micrococcal nuclease (MNase) digestion of purified DNA followed by size selection for nucleosome-sized fragments and subsequent sequencing using the Solexa platform [56]. Control dataset “b” was the product of sonicated purified DNA followed by size selection for nucleosome-sized fragments and sequenced using the Solexa platform [57]. Both control datasets showed a peak of read density at hot spots very similar to the peak of nucleosome occupancy observed in the six sequencing-based nucleosome occupancy maps implying nucleosome occupancy at hot spots, as measured by high throughput sequencing, is likely dominated by experimental artifacts. Because the read density peak was observed in both controls, this bias was most likely not introduced by a MNase sequence preference.

The nucleosome occupancy maps produced using high throughput sequencing show a split peak with a small valley of occupancy centered at hot spots. The low point of this valley is still higher than or equal to the baseline nucleosome occupancy (Figure 4 panels d, e and f). This split peak is likely due to the competing influences of depleted nucleosome density at hot spots with the peak of control read density also centered at hot spots. Thus the trend observed with the sequencing datasets at hot spots is the result of experimental bias as seen in the control datasets combined with nucleosome depletion as seen in the microarray results.

A recent study [21] mapped hot spots and nucleosome occupancy in yeast at high resolution using high throughput sequencing, showing nucleosome depletion at hot spots. Using this dataset we plotted read density at and around hot spots for the MNase and the sonication controls. Similar to the results seen for the Buhler *et al.* hot spots, there is a spurious peak of read density at the Pan *et al.* hot spots (Figure S4). This is likely due to GC content bias, Pan *et al.* hot spots correlate with a higher GC content similar to the Buhler *et al.* hot spots [21]. However, when we plotted nucleosome occupancy at the Pan *et al.* hot spots using the same six sequencing based nucleosome occupancy maps we plotted at the Buhler *et al.* hot spots we observed a valley of nucleosome occupancy centered at hot spots contrary to the peak seen with the Buhler *et al.* hot spots (compare Figure S3 with Figure S5). There is wide variability in the level of bias within the sequencing based nucleosome occupancy datasets examined. This can be seen comparing the distance of the peak height to the baseline in Figure 4 panels d, e and f and Figure S3. The effects of this variability in bias can also be seen when plotting nucleosome occupancy at the Pan *et al.* hot spots (Figure S5). Those datasets with the strongest bias exhibit a strong split peak with depletion centered in the middle of a peak (Figure S5 panel a). Those datasets with a weaker bias show a much smaller split peak (Figure S5 panels c and f).

The Pan *et al.* hot spots are mapped with much higher resolution than the Buhler *et al.* hotspots. A higher fraction of the mapped Pan *et al.* hot spots will be located close to or at the real hot spot, which is likely to be nucleosome depleted; therefore the Pan *et al.* hot spots will have a higher signal to noise ratio than the Buhler *et al.* hot spots. The lower signal to noise ratio of the Buhler *et al.* hot spots is sufficient using microarray based nucleosome occupancy maps, such that the correct biological conclusion can be obtained (Figure 4 panels a, b and c). Using biased nucleosome occupancy maps the lower signal to noise ratio of the Buhler *et al.* hot spots is not sufficient and an incorrect biological conclusions is drawn (Figure 4 panels d, e and f). These same nucleosome occupancy maps, when used with hot spots mapped with much higher resolution and a corresponding greater signal to noise ratio like the Pan *et al.* hot spots can qualitatively produce the correct biological picture (Figure S5).

To further examine this issue using a single sequencing based nucleosome occupancy map, we plotted nucleosome occupancy at three different hot spot datasets: Buhler *et al.* [5], Borde *et al.* [14] and Pan *et al.* [21]. Depending on which hot spot maps were used, nucleosomes were either depleted at hot spots or nucleosome occupancy at hot spots was more difficult to distinguish from baseline (Figure S6 panels d, e and f). Also plotted is a single nucleosome occupancy as mapped by ChIP-chip [55] for the three different sets of hot spots. Contrary to the sequencing based nucleosome occupancy maps, the ChIP-chip based map showed clear nucleosome depletion regardless of which hot spot datasets were used (Figure S6 panels a, b and c). Using high-resolution hot spot datasets coupled with sequencing based nucleosome occupancy maps supports an accurate qualitative interpretation. However, it is quantitatively difficult to determine nucleosome occupancy due to the bias imposed by the sequencing technologies.

It is tempting to conclude that the bias observed at hot spots is due to a GC content bias in next generation sequencing. Our results, in agreement with others [7] demonstrate that hot spots have a tendency to be GC-rich. Several studies have reported evidence of significant GC content bias in next generation sequencing [58], [59], [60], [61]. In support of this hypothesis, plots of nucleosome occupancy near the center of hot spots closely mirror those of GC content (Figure 4, panels d, e and f, and Figure S3).

To further explore this question, read libraries for all six sequencing-based nucleosome occupancy maps plus two control datasets were aligned against the yeast genome, and the GC content of reads that aligned with at least 95% identity (alignment length divided by read length) was calculated. This set was further divided according to whether the reads mapped to intergenic or coding regions (Table 1). An obvious GC bias was discovered in mappable reads (Table 1, column 4). Studies have shown intergenic regions are nucleosome poor compared to coding regions [55], [62]. Since nucleosomes are concentrated to some extent in GC rich coding regions and coding regions are GC-rich a genome-wide examination of sequence bound by nucleosomes would be expected to find a high GC content relative to the genome average. However, it is unlikely that this effect can completely explain the GC bias shown by the six sequencing-based datasets. The GC content in coding regions of the yeast genome is 39.6% whereas that shown by reads mapped to coding regions is ∼42.0%. At 41.3%, the GC content of reads mapped to intergenic regions is much higher than the GC content of intergenic regions (34.8%).

**Table 1:** Average GC content for reads mapped to the yeast genome.

Dataset	Intergenic GC content	Coding GC content	Total GC content
Yeast Genome	34.84%	39.62%	38.15%
Segal 454	42.35%	42.60%	42.40%
Segal Solexa	41.69%	42.20%	41.97%
Pugh 454	41.49%	42.60%	41.81%
Rando Solexa	39.66%	42.26%	41.52%
Friedman Solexa	41.20%	42.99%	42.46%
MacAlpine Solexa	41.71%	42.96%	42.54%
MNase Control	47.84%	47.37%	47.51%
Sonicated Control	38.58%	39.66%	39.19%

Comparison of the GC bias between the two control datasets was particularly interesting. The MNase control showed a strong GC bias in mappable reads of 47.6%, which was nearly 10.0% higher than the overall yeast GC content. The sonication control displayed a much lower GC content bias (39.2%) for mappable reads. All of the sequencing-based nucleosome occupancy maps were produced using MNase digestion. Given the clear GC bias calculated for the MNase control, it is possible that much of the GC bias shown by these maps is a product of MNase cleavage bias. Furthermore, our analysis indicates that the bias seen at hot spots occurs regardless of sequencing platform. Nucleosome occupancy maps produced using both Solexa and 454 sequencing exhibited a bias at recombination hot spots. Given the differing nature of these sequencing platforms, the bias may be introduced during sample preparation and not by the sequencing technologies themselves.

**Figure 6:**
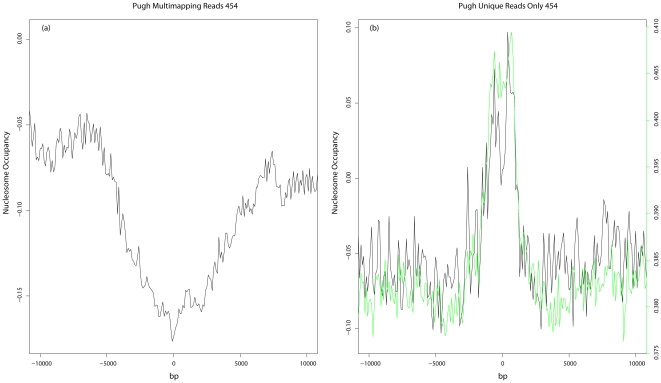
Effect of including multimapping reads. (a) Plot of nucleosome occupancy at hot spots using data produced by the Mavrich *et al*. mapping approach. (b) Plot of nucleosome occupancy of the same dataset at hot spots using uniquely aligned reads only. Green line represents GC content as calculated in Fig. 4.

Not surprisingly, read mapping methodology can also influence downstream analysis. Five of the six sequencing-based datasets and all of the control datasets used only unique aligned reads. However, Mavrich *et al.* [63] used a more lenient mapping approach whereby any alignment yielding an identity less than 90% was discarded and, for alignments between 90% and 95%, only the maximum score was retained [31]. All alignments with greater than 95% identity were kept. The key difference is that their method retained reads that mapped with high confidence to multiple areas along the genome. Using this mapping strategy, a broad shallow valley of read density was observed at hot spots (Figure 6, panel a). When only unique aligned reads from the same dataset were used, a peak of read density similar to that seen with other sequencing-based datasets was seen (Figure 6, panel b). When the control datasets were examined using the Mavrich *et al.* mapping approach, a similar shallow depletion of read density was observed for the sonication control (Figure S7, panel a). The MNase control showed a similar shallow depletion, with the exception of a small peak of read density centered at hot spots. This peak closely mirrors the increase in GC content also centered on hot spots and is likely due to the increased GC bias seen in the MNase control (Table 1). Hence, depending on the mapping approach, opposing biases can be introduced.

Using uniquely aligned reads will bias mapped read density towards unique sequence; including multimapping reads will bias read density towards repetitive sequences. The broad shallow depletion in read density observed at hot spots when allowing multi mapping of reads may reflect the fact that hot spots have a tendency to be located in unique sequences.

Next, we plotted the read density for the six sequencing-based nucleosome occupancy maps following subtraction of the MNase control (see Materials and Methods). When the MNase control was subtracted from the nucleosome occupancy maps, the read density at hot spots is qualitatively in agreement with the microarray-based results, displaying a valley of nucleosome occupancy at hot spots (see Figure S8).

## Discussion

It is difficult using *in silico* analysis alone to demonstrate the existence of a causal relationship between two biological features. What it can do is to identify promising relationships to explore further *in vivo*. Here we have shown that feature selection using machine learning techniques can usefully be applied to a complex biological process. While this manuscript was in preparation a high resolution map of DSB hot spots was published [21]. Sequencing and mapping oligos bound by Spo11 produced this map. Spo11 hot spots compared with hot spots identified by ssDNA hybridization studies such as Buhler *et al.* show a strong degree of concordance with Spo11 hot spots accounting for nearly all hot spots mapped by ssDNA techniques [21].

### Resolution of Hot Spots

The set of DSB hot and cold spots used in this study were derived by mapping single stranded DNA produced by nucleolytic processing of DSBs [5]. These ssDNA fragments may be quite large, 1 to 2 kb. Hence the locations of hot spots as reported by Buhler *et al.* are mapped with some imprecision. This will certainly affect any study that attempts to use this data to elucidate genomic features associated with DSB hot/cold spots.

It is not necessary in this computational analysis for the sites defined as hot spots to exactly overlap the “true” hot spots. It is only necessary that an appropriately sized window centered at the sites defined as hot spots overlap to some degree with the genomic features that are associated with true hot spots. A recent paper studying the association of H3K4me3 with meiotic DSB found enrichment of this mark in a broad region ∼1–2 kb around DSBs [14]. This indicates that regions of high DSB frequency mapped by Buhler *et al.* are likely sufficiently precise to identify at least some chromatin features associated with regions of high meiotic DSBs. Our results strengthen this conclusion of the five features we associated with meiotic DSBs. Three of them H3K4me3, H3K36me3 and GC content have previously been associated with meiotic DSBs. Additionally we obtained a set of recently produced hot spots mapped at high resolution [21] and tested whether the same patterns identified using the low resolution Buhler *et al.* dataset are present using higher resolution data. The same patterns were present using either dataset compare (Figure 3 with Figure S1).

### Mitotic Histone Marks

All of the histone marks associated with recombination in this study were obtained in vegetatively growing mitotic cells. The DSB set we used was mapped in meiotic cells. How can we be sure the histone marks do not change dramatically between these two cell states? There are two major reasons suggesting patterns in histone modifications found at hot spots in mitotic cells may hold true for meiotic cells. First, it has previously been shown that a number of chromatin features present at hot spots in meiotic cells are also present at hot spots in mitotic cells [14], [54]. For example, H3K4me3 does not change dramatically in mitotic compared to meiotic cells [14]. The set of hot spots mapped in meiotic cells by Buhler *et al.* have been shown to be on average nucleosome depleted in mitotic cells [54] indicating that at least two chromatin features associated with recombination hot spots in meiotic cells are also present to some degree at those same sites in mitotic cells. Additionally, a recent study examined the changes in chromatin states from mitotic to meiotic cells for a number of nucleosome associated biological features including H3K9ac, H3K4, H3K36 and H3K79 tri-methylation. The conclusion reached was that histone modification states were remarkably stable changing little between mitotic and meiotic cells [64]. These authors also examined the distribution of H3K36me3, H3K4me3 and H3K79me3 at hot and cold spots in meiotic cells. Their results mirror our own obtained in mitotic cells. In addition, Zhang *et al.* showed that in general the distribution of these marks change little between mitotic to meiotic cell states suggesting that the chromatin features associated with hot or cold spots are present in both mitotic and meiotic cells.

Second, we show that, in general, histone modifications peak heights for H3K14ac and H3K4me3 found in promoter regions of genes with hot spots are proportional to the strength of the corresponding hot spots and not dependent on transcriptional rates. The fact that this pattern is present in mitotic cells is strongly suggestive it will be present in meiotic cells. Our results showing an association between DSB frequencies measured in meiotic cells and enrichment for histone modifications measured in mitotic cells suggests that nucleosome occupancy and H3K4me3 may not be the only chromatin features that mark sites of meiotic DSBs in mitotic cells before the entrance to meiosis. Although, this is a question that cannot be answered by *in silico* analysis because it requires further experimentation measuring the distribution of these marks for both meiotic and mitotic cells.

### Role of Histone Modifications

The role of histone modifications in specifying sites of Spo11-catalyzed DSBs is unclear. Specific marks could serve to directly recruit proteins involved in recombination. Alternatively, histone modifications, such as acetylation, may act indirectly by modifying the local chromatin structure. Histone acetyltransferases and ATP-dependent chromatin remodeling factors have been shown to regulate recombination at the *ade6-M26* hotspot in S. *pombe* [15], [65]. Deletion of the histone acetyltransferase *GCN*5 gene causes a significant delay in chromatin remodeling, leading to a partial reduction in recombination frequency. Double deletion of SNF22, a component of a chromatin remodeling complex, and GCN5 leads to a complete loss of meiotic recombination. RSC4p, a component of the chromatin remodeling complex RSC, contains tandem bromodomains that recognize H3K14ac, suggesting that this mark may recruit chromatin remodeling factors directly [66]. In addition, acetylation leads to a more open and less condensed chromatin structure, allowing easier access for recombination proteins or chromatin remodeling complexes.

Dot1p the methyltransferase responsible for lysine 79 methylation has been linked with DNA repair [67]. Deletion of Dot1p confers increased sensitivity to radiation in yeast [68]. Additionally the correct function of the DNA checkpoint response requires H3 methylation by Dot1p [69] . The presence of Dot1p is necessary for efficient repair of DSB by sister chromatid repair [70]. This suggests H3K79me3 may be associated with regions of low meiotic DSBs frequency because it is a marker for DNA repair.

Another possibility is that specific histone modifications may affect DSB frequencies indirectly by inhibiting or enhancing other histone modifications that play a more direct role. For instance, preventing H2B ubiquitination leads to decreased meiotic DSBs[35]. By promoting H3K4me3, H2B ubiquitination may be enhancing DSB formation [71]. Another possible example of similar “cross-talk” between histone modifications is H3K36me3-mediated repression of DSB formation at the well-studied *HIS4* recombination hot spot in budding yeast [44]. H3K36me3 recruits the Rpd3 histone deacetylase [45], suggesting that this mark may have an indirect negative effect on DSB frequency by preventing or reducing histone acetylation since there appears to be a positive correlation between histone acetylation and DSB frequency at some hot spots [15].

### Nucleosome mapping

Locke et al. [56] were able to predict nucleosome positions using nuclesome free control data they suggest this could be because MNase sequence preference or sonication fragmentation coincides with nucleosome excluding sequence. If this were the case any “peaks” of read density in the MNase or sonication control datasets at hot spots may well reflect true nucleosome occupancy. In support of this hypothesis a recent study in mice found evidence of increased nucleosome binding at hot spots [72].

We do not think this is the case for the genomic loci in question for a number of reasons. One the same set of genomic loci used in our study i.e.(Buhler *et al.* Hot spots) were recently shown to be on average nucleosome depleted using FAIRE [54]. This directly contradicts the sequencing based results at these same loci (Figure 4 panels d, e and f). Two microarray based nucleosome occupancy maps are in agreement with one another but disagree with the results of the uncorrected sequencing based studies (Figure 4). Finally a number of individual hot spots have been examined (see above) and in general they are nucleosome depleted.

The extent to which nucleosome binding is based on sequence preferences is currently an active area of research [23], [57]. One approach to answering this question is comparing nucleosome maps produced *in vitro* and *in vivo* [23]. Our results, along with others [73], [74], indicate that a systematic bias can dominate at certain genomic loci, thereby obscuring the true biological representation. It is unknown to what extent this influences the genome wide similarity observed between *in vivo* and *in vitro* produced nucleosome occupancy maps.

Using control experiments to remove the systematic bias is an obvious approach in dealing with experimental artifacts. Unfortunately, producing suitable controls is not necessarily straightforward [75]. Previously, controls have rarely been used in nucleosome mapping with high throughput sequencing methods. When experimental bias is not controlled for, the opposite of the most likely correct biological picture is observed at yeast meiotic hot spots mapped at low resolution. However, when we subtract a MNase control experiment from the nucleosome occupancy maps, the correct biological interpretation can be derived indicating the suitability of this control for the loci under investigation in this study. Furthermore, our results underscore the importance of addressing experimental bias in nucleosome mapping high throughput sequencing experiments. Our analysis is not intended to be a comprehensive examination of all possible biological features potentially associated with meiotic DSB frequency Future work could expand the set of genome wide features being examined at sites of high/low meiotic DSB frequencies. Here we have shown feature selection can productively be used to identify promising biological associations. Our approach successfully identified previously known correlations while making several novel predictions.

## Supporting Information

Figure S1 **Plots of average modification level around transcription start sites (TSS) using Pan** ***et al.*** **hot spots.** Figure is produced as described for Figure 3 with one difference. For panels a, b, d and e genes with a hotspot in their promoter regions were further divided based on the strength of the hot spot. The blue line is the given histone modification plotted upstream of genes whose hot spot is below the first quartile. The red line is genes whose hot spot strength falls between the first and second quartile. The purple line is genes whose hot spots falls betweeen the second and third quartile. The green line is genes whose hot spots strength is greater than the third quartile.
(TIF)

Figure S2 **Gene expression comparison in meiotic cells.** Panels a-d is comparing gene expression between genes associated with hot spots to genes not associated with hot spots. Height of bars represents the difference in median gene expression for genes associated with hot spots to genes not associated with hot spots (i.e. Median hot gene expression – Median not hot gene expression). Time points represent time after yeast culture is placed in sporulating media. Panels (a) an (c) represent gene expression measured at the given time points for sporulation deficient SK1 and W303 strains these strains do not enter meiosis. Panels (b) and (d) represent gene expression for sporulation-proficient SK1 and W303 strains. An asterisk represents the difference in medians is significant with p-value<0.05, p-value calculated using the Wilcox rank sum test. Panels c-h is as described above except height of bars represents the difference in median gene expression for genes associated with cold spots to genes not associated with cold spots (i.e. Median cold gene expression – Median not cold gene expression). Gene expression is represented by hybridization fluorescence intensities.
(TIF)

Figure S3 **Nuclesome occupancy at Buhler** ***et al.*** **hot spots for all sequencing-based datasets.** For all datasets, reads were mapped to the yeast genome. Only uniquely aligned reads were retained and the count mapped to each base pair was calculated. The z-score standardized count of reads is plotted using the same procedure as described for Figure 4 with the green line representing GC content. (references for datasets: a [76], b [23], c [77] , d [78], e [63] and f [79].
(TIF)

Figure S4 **Read density for sequencing controls at Pugh** ***et al.*** **hot spots.** (a) Purified DNA digested with micrococcal nuclease (MNase) and sequenced using the Solexa platform. (b) Purified DNA following sonication and sequencing using the Solexa platform. The black line indicates the z-score standardized mapped read density. Data was smoothed using loess smoothing.
(TIF)

Figure S5 **Nucleosome occupancy at Pugh** ***et al.*** **hot spots for all sequencing-based datasets.** For all datasets, reads were mapped to the yeast genome. Only uniquely aligned reads were retained and the count mapped to each base pair was calculated. The z-score standardized count of reads is plotted at centered Pugh *et al* hot spots. Plot is produced similar to Figure 4 and Figure S3. (References for datasets: a [76], b [23], c [77] , d [78], e [63] and f [79].
(TIF)

Figure S6 **Nucleosome occupancy at recombination hot spots obtained at various resolutions.** Z-score standardized nucleosome occupancy is shown in 100 bp bins (y-axis). The center of the aligned hot spots is zero on the x-axis. Panels a, b and c represent nucleosome occupancy data measured by ChIP-chip produced by Lee *et al.* [55] at three different hot spot datasets from left to right [5], [14], and [21]. Panels d, e and f represent nucleosome occupancy in the same three datasets but now using a nucleosome occupancy map produced by ChIP-seq [64]. This sequencing based nucleosome occupancy map has previously been used in analyzing nucleosome occupancy at hot spots as defined by Borde *et al.* [14].
(TIF)

Figure S7 **Sonication and MNase control plotted at Buhler** ***et al.*** **hot spots allowing multimapping reads.** Reads for sonicated (a) and MNase-digested controls (b) were mapped allowing multimapping of reads. Read density centered at hot spots is plotted. Data was smoothed using loess smoothing.
(TIF)

Figure S8 **Nuclesome occupancy at Buhler** ***et al.*** **hot spots for all sequencing-based datasets following subtraction of the MNase control.** Nucleosome occupancy was plotted at hot spots for all sequencing-based nucleosome mapping datasets following subtraction of the MNase control as described in the text. Data plotted similarly to Figure 4.
(TIF)
