# Supplementary figures and images for: Analysis of Biological Features Associated with Meiotic Recombination Hot and Cold Spots in Saccharomyces cerevisiae
Source: PLoS One. 2011 Dec 29;6(12):e29711. doi: 10.1371/journal.pone.0029711 (PMC3248464; doi:10.1371/journal.pone.0029711)

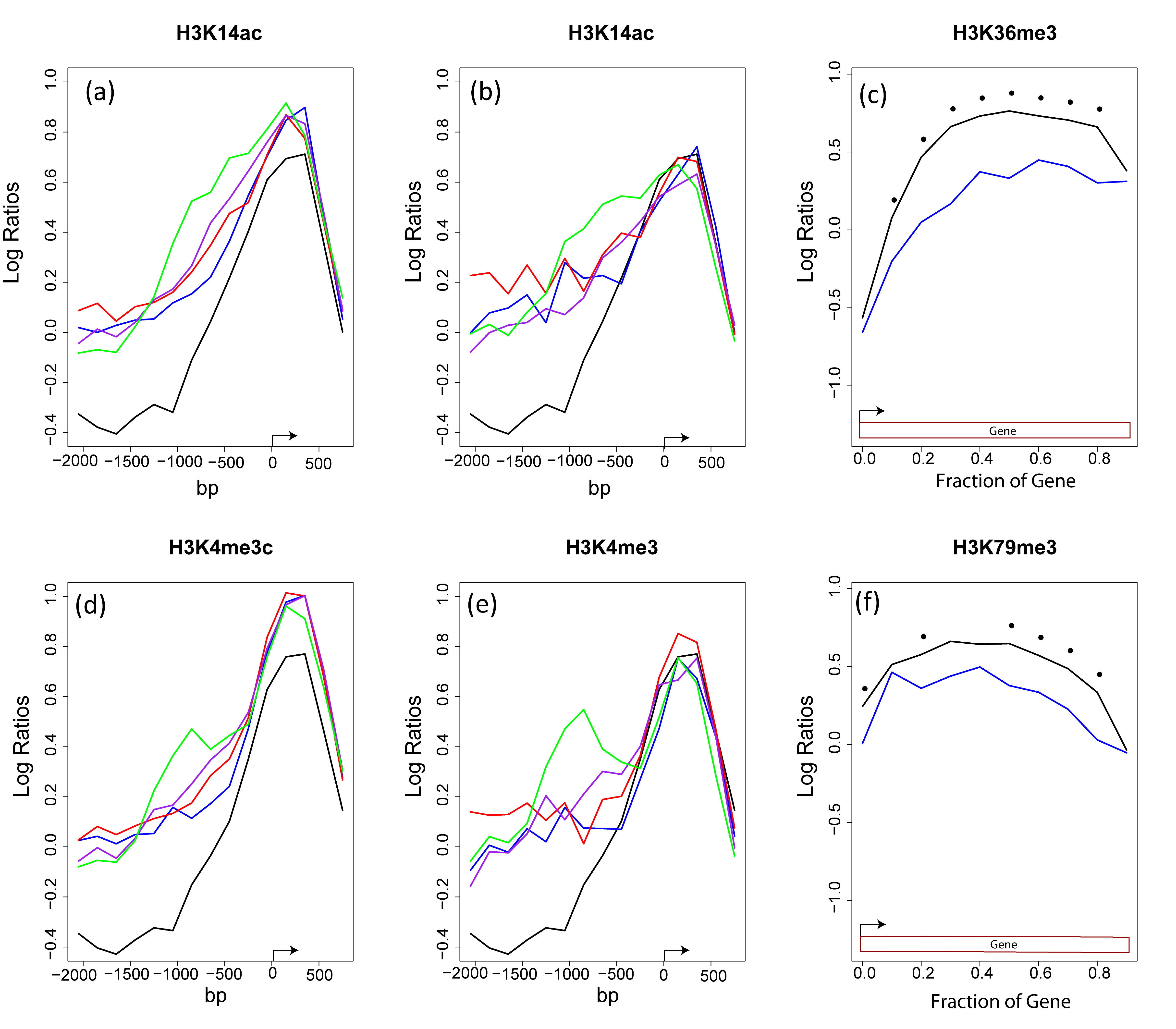

Supplement: Figure S1 — Plots of average modification level around transcription start sites (TSS) using Pan et al. hot spots. Figure is produced as described for Figure 3 with one difference. For panels a, b, d and e genes with a hotspot in their promoter regions were further divided based on the strength of the hot spot. The blue line is the given histone modification plotted upstream of genes whose hot spot is below the first quartile. The red line is genes whose hot spot strength falls between the first and second quartile. The purple line is genes whose hot spots falls betweeen the second and third quartile. The green line is genes whose hot spots strength is greater than the third quartile. (TIF) [file pone.0029711.s001.tif]

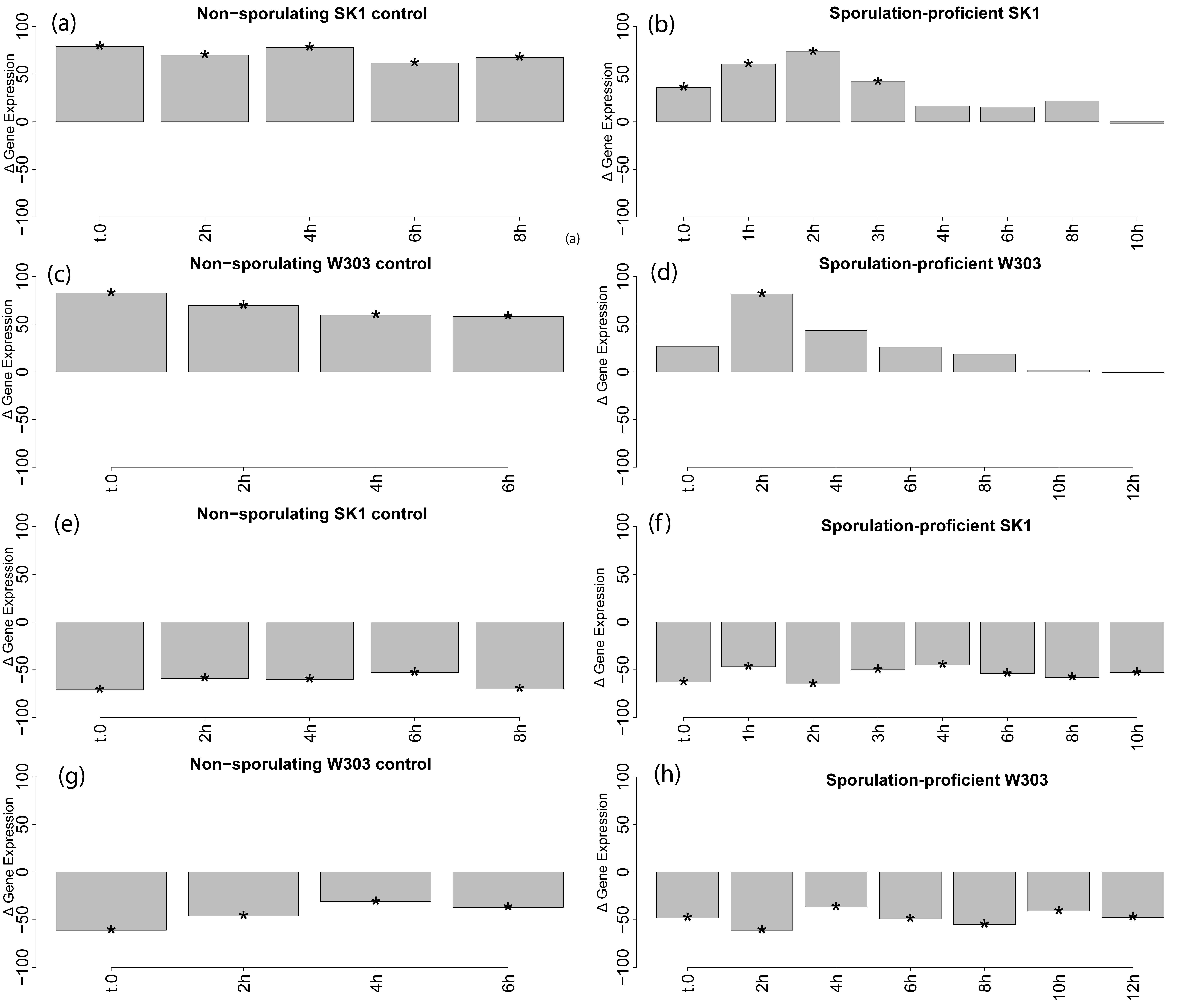

Supplement: Figure S2 — Gene expression comparison in meiotic cells. Panels a-d is comparing gene expression between genes associated with hot spots to genes not associated with hot spots. Height of bars represents the difference in median gene expression for genes associated with hot spots to genes not associated with hot spots (i.e. Median hot gene expression – Median not hot gene expression). Time points represent time after yeast culture is placed in sporulating media. Panels (a) an (c) represent gene expression measured at the given time points for sporulation deficient SK1 and W303 strains these strains do not enter meiosis. Panels (b) and (d) represent gene expression for sporulation-proficient SK1 and W303 strains. An asterisk represents the difference in medians is significant with p-value<0.05, p-value calculated using the Wilcox rank sum test. Panels c-h is as described above except height of bars represents the difference in median gene expression for genes associated with cold spots to genes not associated with cold spots (i.e. Median cold gene expression – Median not cold gene expression). Gene expression is represented by hybridization fluorescence intensities. (TIF) [file pone.0029711.s002.tif]

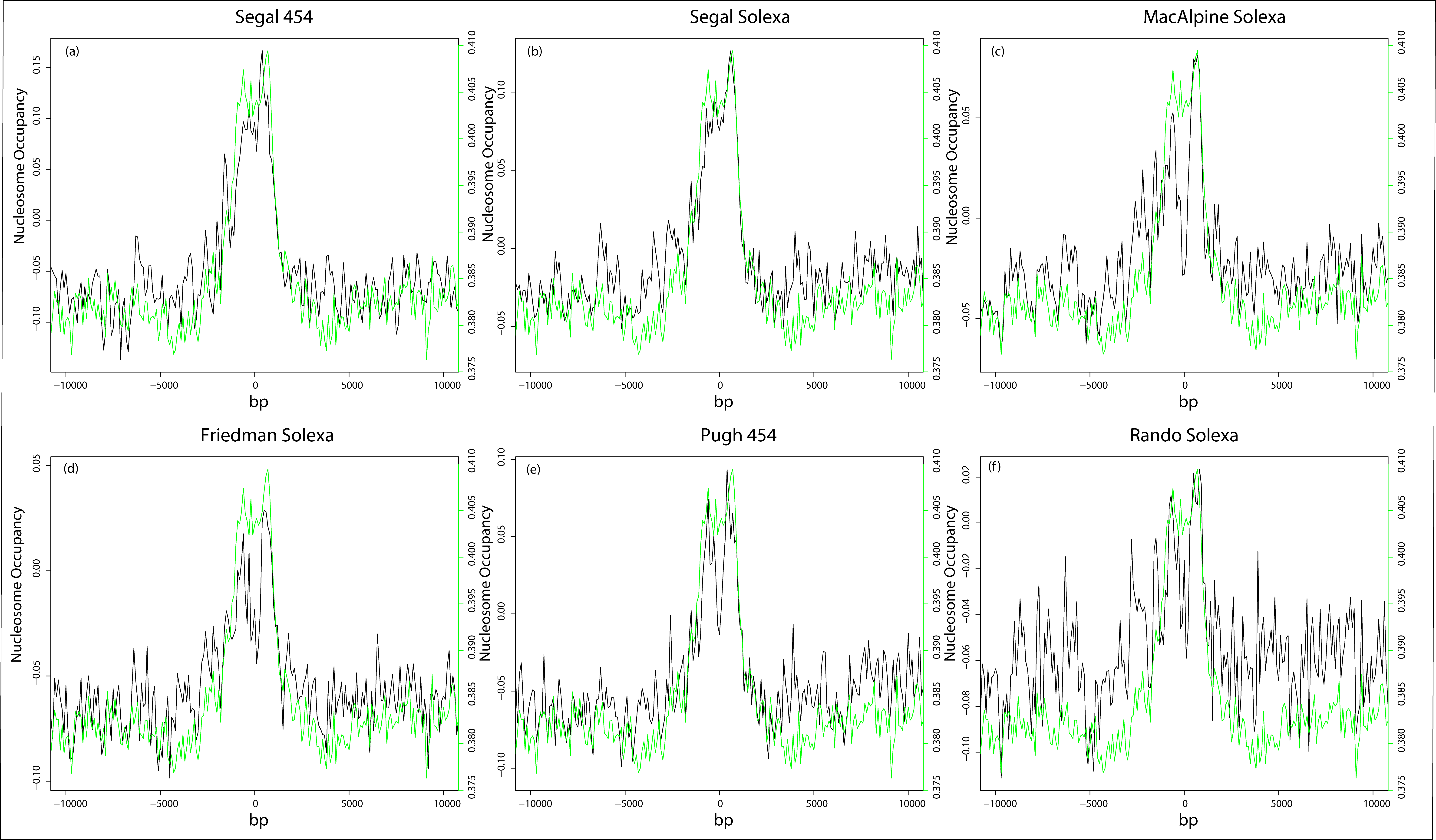

Supplement: Figure S3 — Nuclesome occupancy at Buhler et al. hot spots for all sequencing-based datasets. For all datasets, reads were mapped to the yeast genome. Only uniquely aligned reads were retained and the count mapped to each base pair was calculated. The z-score standardized count of reads is plotted using the same procedure as described for Figure 4 with the green line representing GC content. (references for datasets: a [76], b [23], c [77] , d [78], e [63] and f [79]. (TIF) [file pone.0029711.s003.tif]

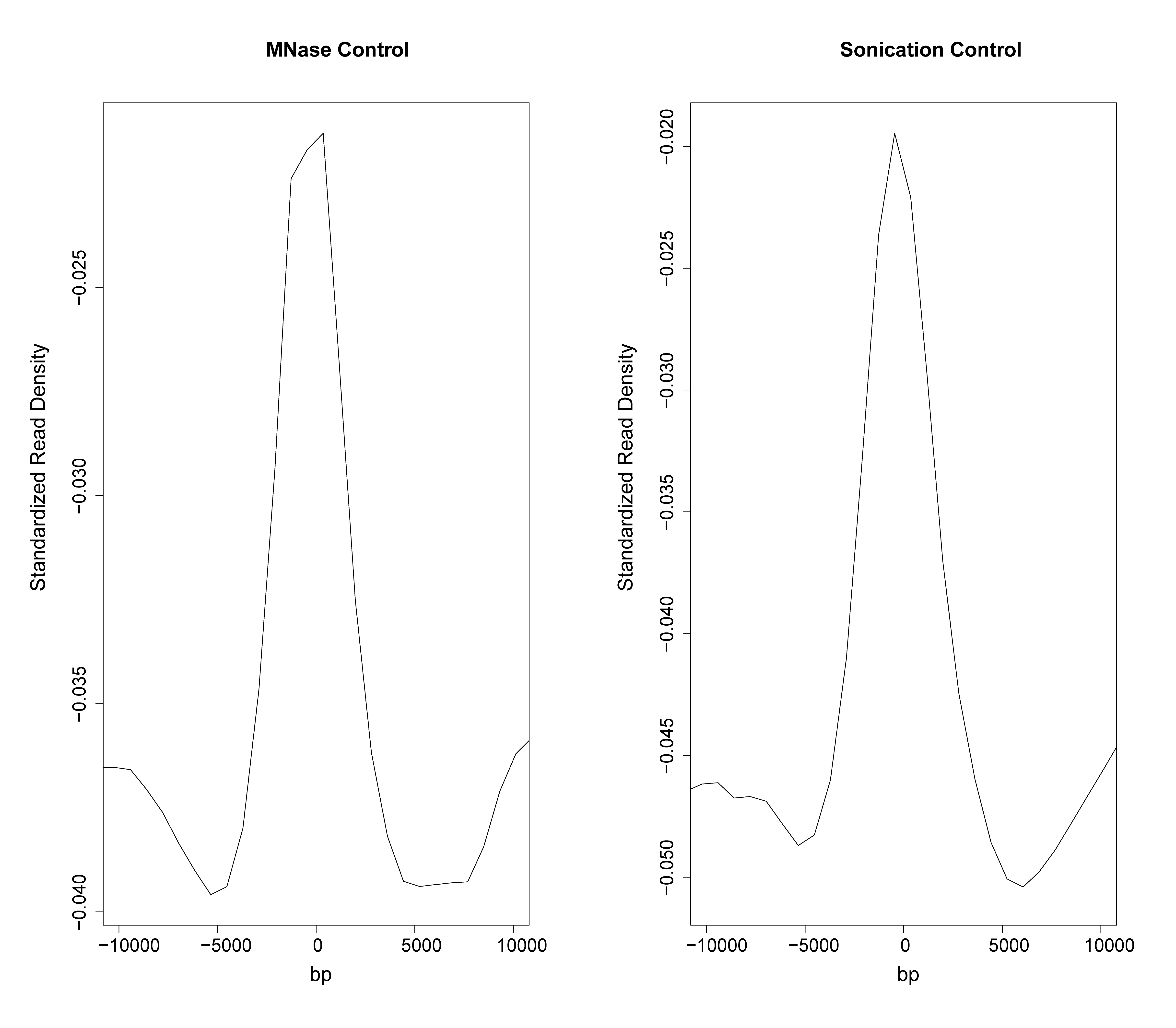

Supplement: Figure S4 — Read density for sequencing controls at Pugh et al. hot spots. (a) Purified DNA digested with micrococcal nuclease (MNase) and sequenced using the Solexa platform. (b) Purified DNA following sonication and sequencing using the Solexa platform. The black line indicates the z-score standardized mapped read density. Data was smoothed using loess smoothing. (TIF) [file pone.0029711.s004.tif]

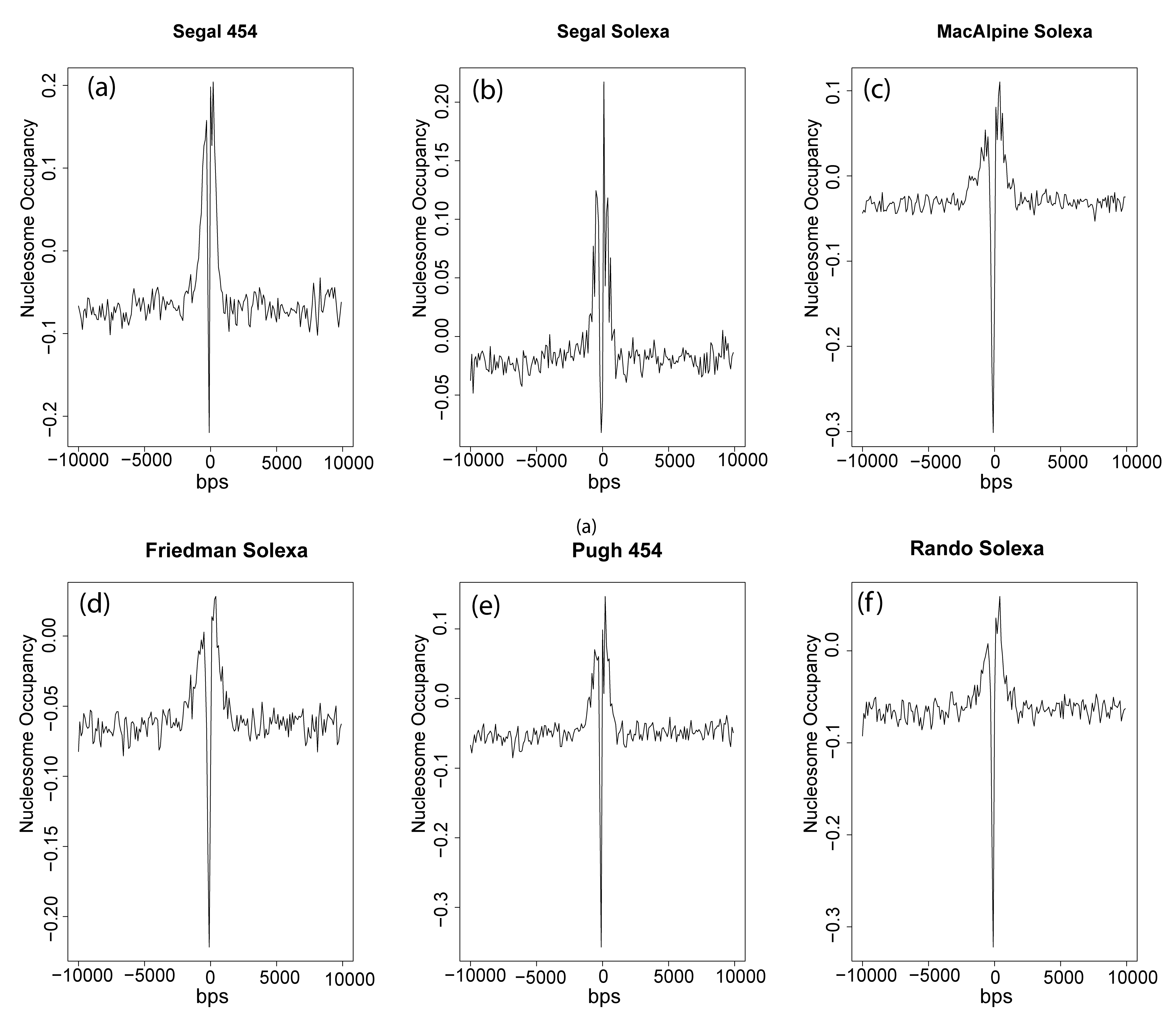

Supplement: Figure S5 — Nucleosome occupancy at Pugh et al. hot spots for all sequencing-based datasets. For all datasets, reads were mapped to the yeast genome. Only uniquely aligned reads were retained and the count mapped to each base pair was calculated. The z-score standardized count of reads is plotted at centered Pugh et al hot spots. Plot is produced similar to Figure 4 and Figure S3. (References for datasets: a [76], b [23], c [77] , d [78], e [63] and f [79]. (TIF) [file pone.0029711.s005.tif]

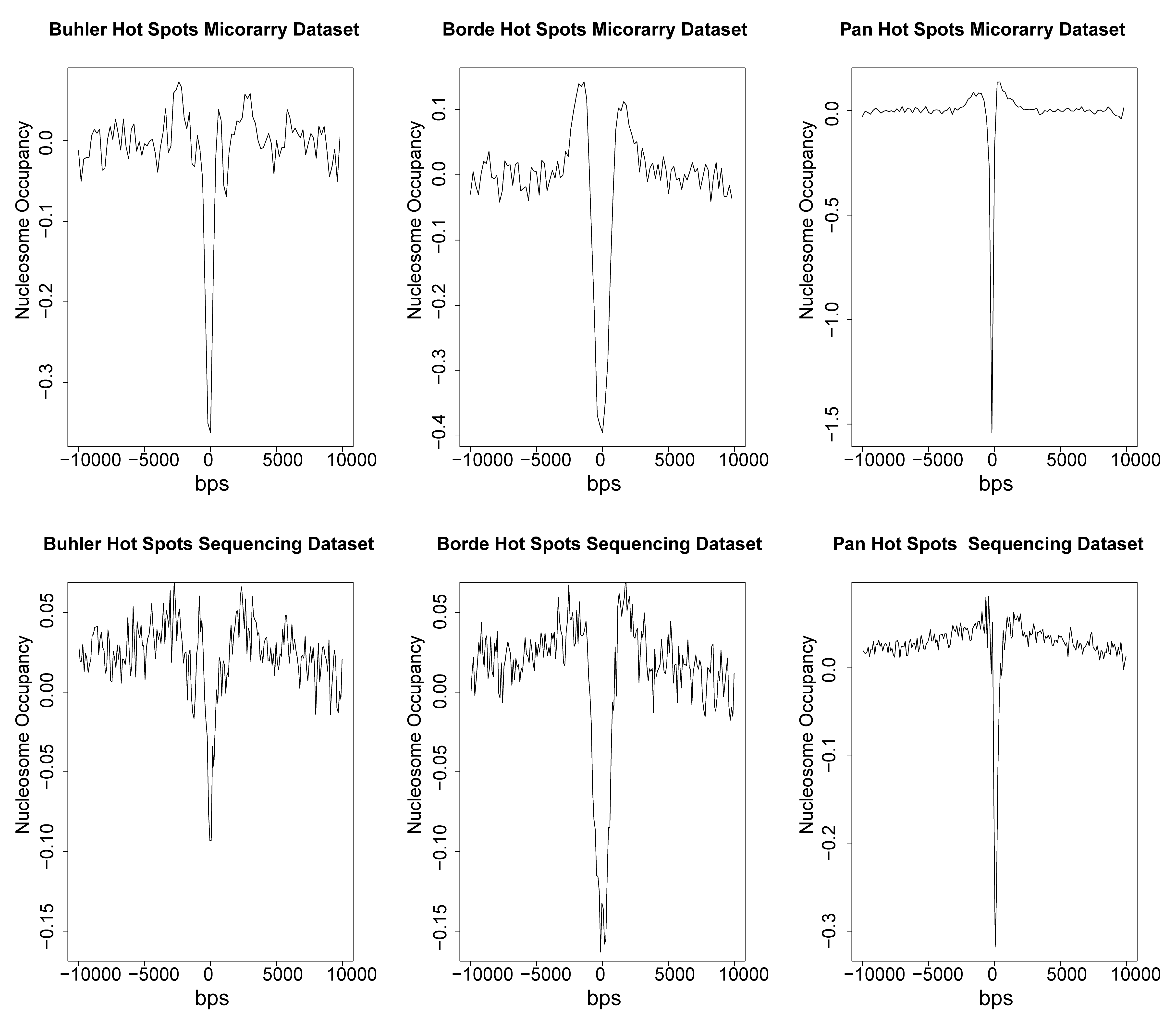

Supplement: Figure S6 — Nucleosome occupancy at recombination hot spots obtained at various resolutions. Z-score standardized nucleosome occupancy is shown in 100 bp bins (y-axis). The center of the aligned hot spots is zero on the x-axis. Panels a, b and c represent nucleosome occupancy data measured by ChIP-chip produced by Lee et al. [55] at three different hot spot datasets from left to right [5], [14], and [21]. Panels d, e and f represent nucleosome occupancy in the same three datasets but now using a nucleosome occupancy map produced by ChIP-seq [64]. This sequencing based nucleosome occupancy map has previously been used in analyzing nucleosome occupancy at hot spots as defined by Borde et al. [14]. (TIF) [file pone.0029711.s006.tif]

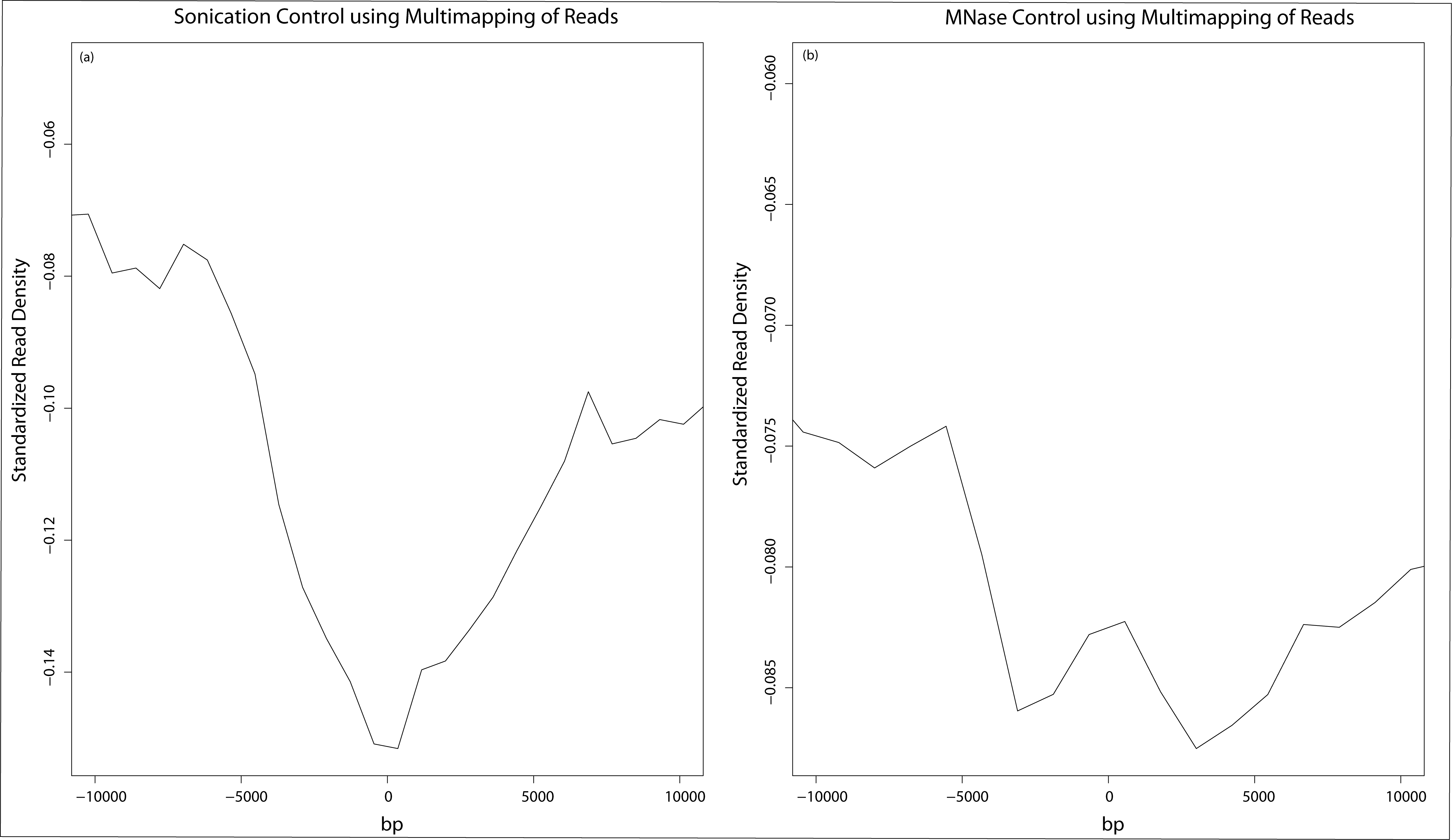

Supplement: Figure S7 — Sonication and MNase control plotted at Buhler et al. hot spots allowing multimapping reads. Reads for sonicated (a) and MNase-digested controls (b) were mapped allowing multimapping of reads. Read density centered at hot spots is plotted. Data was smoothed using loess smoothing. (TIF) [file pone.0029711.s007.tif]

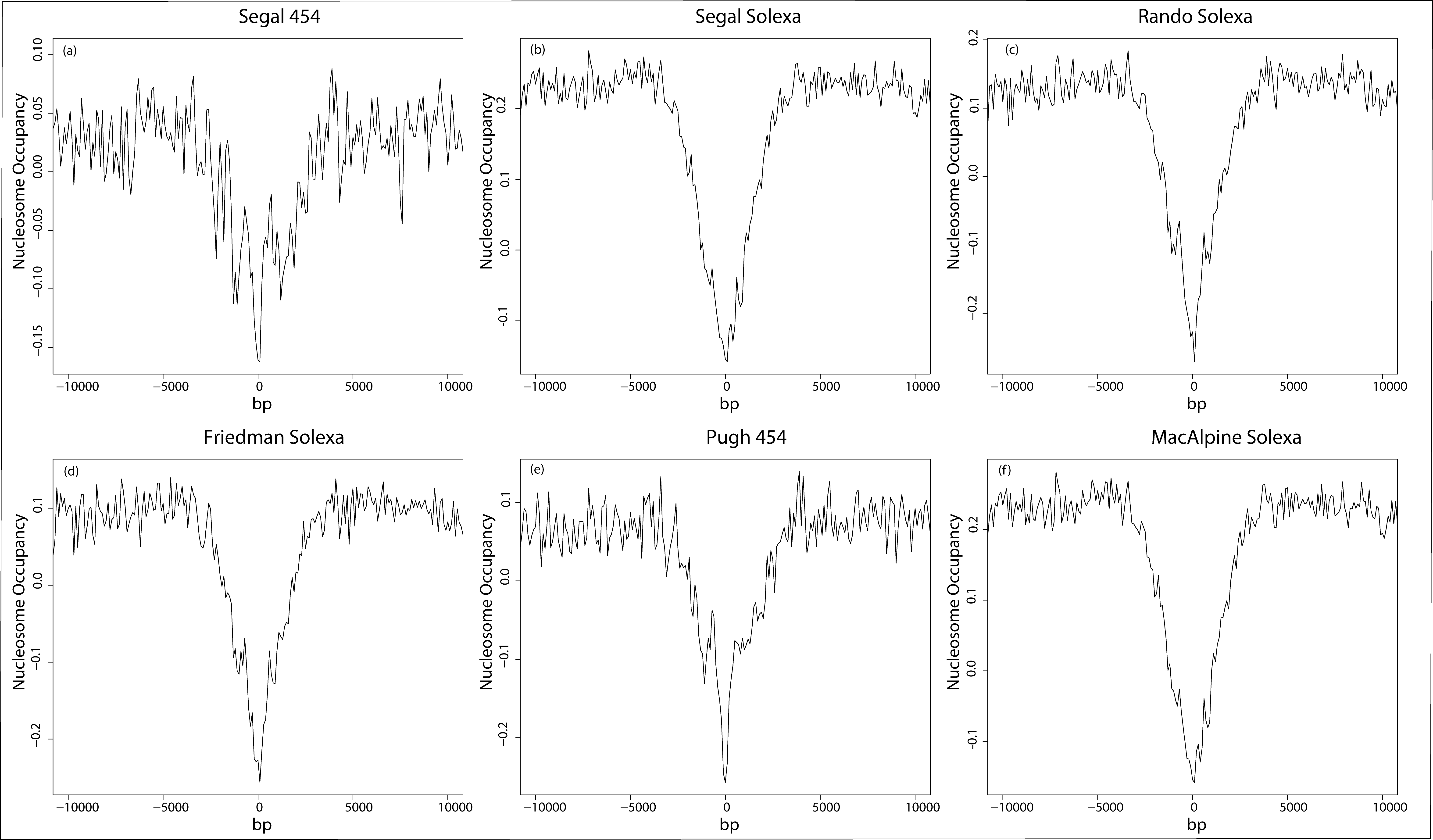

Supplement: Figure S8 — Nuclesome occupancy at Buhler et al. hot spots for all sequencing-based datasets following subtraction of the MNase control. Nucleosome occupancy was plotted at hot spots for all sequencing-based nucleosome mapping datasets following subtraction of the MNase control as described in the text. Data plotted similarly to Figure 4. (TIF) [file pone.0029711.s008.tif]
